# Repetitive administration of rituximab can achieve and maintain clinical remission in patients with MCD or FSGS

**DOI:** 10.1038/s41598-023-32576-7

**Published:** 2023-04-28

**Authors:** Thomas Osterholt, Polina Todorova, Lucas Kühne, Rasmus Ehren, Lutz Thorsten Weber, Franziska Grundmann, Thomas Benzing, Paul Thomas Brinkkötter, Linus Alexander Völker

**Affiliations:** 1 Department II of Internal Medicine and Center for Molecular Medicine Cologne, Faculty of Medicine, University Hospital of Cologne, University of Cologne, Cologne, Germany; 2 Faculty of Medicine, Pediatric Nephrology, Children’s and Adolescents’ Hospital, University Hospital of Cologne, University of Cologne, Cologne, Germany; 3 Cologne Excellence Cluster on Cellular Stress Responses in Aging-Associated Diseases, University of Cologne, Cologne, Germany

**Keywords:** Glomerular diseases, Focal segmental glomerulosclerosis, Minimal change disease

## Abstract

Minimal change disease (MCD) and focal segmental glomerulosclerosis (FSGS) are glomerulopathies associated with nephrotic syndrome. Primary forms of these diseases are treated with various regimes of immunosuppression. Frequently relapsing or glucocorticoid-dependent courses remain challenging. Here, a B-cell-depleting strategy with rituximab represents a salvage option although data are sparse in the adult population. In particular, there is limited evidence on the efficacy of restoring remission after initial successful treatment with rituximab and whether patients benefit from an individualized, relapse-based approach. We identified 13 patients who received multiple therapies with rituximab from the FOrMe-registry (NCT03949972), a nationwide registry for MCD and FSGS in Germany, or from the University Hospital of Cologne. Disease status, changes in serum creatinine, proteinuria, and time to relapse were evaluated. Relapse-free survival was compared to the patients’ previous therapy regimens. Through all treatment cycles, an improvement of disease activity was shown leading to a complete remission in 72% and partial remission in 26% after 3 ($p<$0.001) and 6 months ($p<$0.001). Relapse-free survival increased from 4.5 months (95%-CI 3–10 months) to 21 months (95%-CI 16–32 months) ($p<$0.001) compared to previous immunosuppression regimens with no loss in estimated glomerular filtration over time (*p* = 0.53). Compared to continuous B-cell depletion, an individualized relapse-based approach led to a reduced rituximab exposure and significant cost savings. Relapse-based administration of rituximab in patients with MCD/FSGS with an initial good clinical response did not result in a decreased efficacy at a median follow-up duration of 110 months. Thus, reinduction therapies may provide an alternative to continuous B-cell-depletion and reduce the long-term side effects of continuous immunosuppression.

## Introduction

Minimal change disease (MCD) and focal segmental glomerulosclerosis (FSGS) are both common glomerulopathies although their absolute incidences are low at 0.8/100 000/year^1^. It is critical to understand both MCD and FSGS as histopathologic patterns of glomerular and primarily podocyte injury that can be caused by various gene defects, underlying systemic diseases, and conditions as well as drugs and toxins^2^. MCD is the most common pediatric glomerulopathy but also accounts for 10–15% of nephrotic syndromes (NS) in adults^3^. In contrast, FSGS is a glomerulopathy primarily in adults with an increasing incidence worldwide^1^. Both diseases can be classified into primary, secondary, and genetic variants, and some authors see a disease continuum of MCD to FSGS ^2,4^. In the pediatric population, these diseases are classified as primary and secondary; primary forms also include genetic variants. The distinction between primary and secondary forms is based on clinical features including the presence of nephrotic syndrome and electron microscopy indicating the extent of foot process effacement^5^. In MCD, nephrin autoantibodies have been shown to correlate with disease activity in a subset of patients^6^. Primary FSGS is most likely caused by presumed circulating soluble factors^2^. Soluble urokinase plasminogen activator receptor (suPAR) has been studied intensively in this context^7–9^. Although suPAR levels are elevated in various other diseases, recent observations showed that suPAR levels are higher in patients with FSGS compared to other glomerular disease, and increased levels of suPAR are associated with progression to ESKD^10^. Additionally, in patients with FSGS and high levels of suPAR, rituximab was shown to be ineffective^11^. suPAR, together with other FSGS risk factors, may increase the pathogenicity of glomerular disease, for example by cooperating with a risk variant version of Apolipoprotein L1 (APOL1)^12,13^. Other, less studied candidates are cardiotrophin-like cytokine factor 1 (CLCF1) and anti-CD-40 antibodies.

Broad immunosuppression represents the cornerstone of therapy for primary forms of MCD and FSGS with different response patterns. Steroids are most commonly used as induction therapy. Secondary therapies comprise calcineurin inhibitors such as cyclosporine (CsA) or tacrolimus (Tac), mycophenolate mofetil (MMF), cyclophosphamide (CYC) and rituximab (RTX)^14^. RTX in the treatment of MCD confers a complete remission rate of 74.7% (95% CI 62.5–84.0%) and a partial remission in 5.6% (95% CI 9.9–24.8%) of cases while 36% relapse in the follow-up. Patients with FSGS show overall remission rates of 53.6% (95% CI 15.8–87.6%) with a complete remission of 42.9% (95% CI 10.8–82.3%) and a partial remission rate of 10.7% (95% CI 7.0–59.2%)^15^.

Long-term treatment with RTX has been well-tolerated in patients with auto-immune disease such as rheumatoid arthritis^16^. Its repetitive use has been described in patients with refractory and relapsing systemic lupus erythematosus^17^ and ANCA vasculitis^18–20^. Recently Shiha et al. showed that sequential therapies with RTX in pediatric nephrotic syndrome lead to a decreased relapse rate and a median remission of two years in the pediatric population but were associated with a high rate of adverse events mostly infusion reactions or infections but also 10.8% serious adverse events^21^. Chan et al. evaluated different RTX regimen in pediatric patients with steroid-dependent or frequently-relapsing nephrotic syndrome. Depending on the dosage of RTX and concomitant maintenance therapy, the relapse-free survival varied from 8.5 to 14.3 months with a reported adverse event rate of 16%, which consisted mostly of infusion-related adverse events^22^. In the CNI-dependent pediatric population, Ravani et al. reported a relapse-free survival of 5.6 months for the first RTX administration and 8.5 for further administrations of RTX with mostly infusion-related and no severe side effects^23^.

In the pediatric nephrologic community, there is a trend towards an earlier use of RTX in the treatment of nephrotic syndrome. While glucocorticoids remain the initial therapeutic approach, relapsing or resistant forms are treated with MMF, CNI, CYC, RTX, levamisole, or combinations, and there is an increasing body of literature for RTX showing non-inferiority or even superiority compared to commonly used immunosuppressant drugs. A single intravenous infusion of RTX in low steroid-dose frequently relapsing nephrotic syndrome (FRNS) and steroid-dependent nephrotic syndrome (SDNS) provided a benefit in relapse-free survival without steroids in children^24^. Non-inferiority or even higher rates of relapse-free survival have been associated with the use of RTX compared to other second line immunosuppression such as CNI or CYC^25–28^.

In the adult population, there is also increasing evidence for the use of RTX in MCD and FSGS but significant relapse rates, as stated before, remain an issue^15^. While various treatment regimens have been described for the first administration of RTX in MCD and FSGS, there is little information about further treatment after the initial successful therapy with RTX. For those patients, especially for those who do not tolerate other immunosuppressant medication, long-term treatment strategies with RTX are needed.

Therapeutic approaches in MCD and FSGS to RTX treatment can be summarized mainly as continuous B-cell depletion as part of maintenance therapy or reinduction therapy after disease relapse. In the first case, RTX is administered at a fixed interval, either every 3 to 6 months^29–34^ or B-cell-guided based on clinical needs^35–37^. The verdict whether either one of these strategies is preferable is still out. Whether the initial effect of RTX induction therapy can be reproduced later in subsequent reinduction therapies as an alternative to continuous B-cell depletion with fixed intervals and/or guided by CD19-positive cell counts remains unclear. Especially in adult patients, there is little evidence to sequential therapies compared to children^21,22^. Here, we add to the body of literature, evaluating the repetitive administration of RTX in adult patients with MCD and FSGS for induction and maintenance over a long period.

## Methods

To further substantiate the role of RTX in the treatment of relapsed MCD or FSGS, we performed a retrospective analysis of patients with an informed written consent in the German MCD/FSGS registry (FOrMe registry (NCT03949972)^38^) and mostly treated at the University Hospital of Cologne between 2010 and 2021, a central european tertiary care center with a catchment area of about 5,000,000 inhabitants. Approval was obtained from the Institutional Ethics Committee of the University Hospital Cologne (No. $21-1013\_1$ and No. $21-1568-retro$). The study was conducted in accordance with the Declaration of Helsinki and the good clinical practice guidelines of the International Conference on Harmonization. All adult ($\ge$ 18 years) patients with biopsy-proven primary MCD or FSGS from the FOrMe-registry or the University Hospital of Cologne were included if they had received at least two cycles of RTX between 2010 and 2021. A therapy cycle hereby was defined as a single dose or multiple consecutive doses (up to 4×375 $\hbox {mg/m}^{2}$ body surface area one week apart) of RTX administered for induction of remission or in one case for the initial switching from previous immunosuppression in an CNI-dependent patient who experienced side effects from the latter. The dosage and number of administrations was at the discretion of the treating physician. The previous immunosuppression could be continued or withdrawn by the treating physician after the administration of RTX.

Previous immunosuppressant medication was assessed. Laboratory values were extracted from our database. Proteinuria was measured in mg/d and mg/g creatinine and were used equivalently. Negative dipstick-results were also adjudicated for documenting remission. B-cell count was not routinely measured during therapy.

Classification as relapse of nephrotic syndrome (NS), partial remission (PR), or complete remission (CR) was extracted or made according to all available data in our database. Relapse, partial remission, and complete remission were defined in accordance to the KDIGO Guidelines 2012 which applied at the time. Serum albumin was not routinely measured if the proteinuria was $\le$ 3500 mg/g creatinine and not included in the definition.

Time to relapse was calculated from the beginning of first administration of RTX in the last cycle. To compare the efficacy of RTX with previous treatment regimens of those patients, documented time to relapse before the first administration of RTX was assessed and compared to the time to relapse after the use of RTX. Subgroup analyses were performed in patients who received maintenance therapy with either MMF or CNI after the administration of RTX and in patients with FSGS and MCD. In a case with primary steroid resistance, we evaluated this treatment response to glucocorticoids as relapse at month zero.

### Statistical analysis

For statistical analysis we used R Statistics 4.1^39^ with additional packages *dplyr* for data-wrangling^40^, *ggplot2* for plotting visualization^41^, *lubridate* for datetime calculations^42^, *psych-package* for descriptive data^43^ and *survival*^44^ for survival analyses. The test for normal distribution was performed using the Shapiro test. Categorial data were analyzed using the Fisher’s exact test. Ordinal data were compared using the Wilcoxon rank sum test and paired ordinal data were compared using the Wilcoxon signed rank test. Time to relapse was analyzed using the cox-proportional-hazard and Kaplan-Meier estimators. All group comparisons were two-sided with a significance level of $\alpha =0.05$. Subgroup analyses were performed comparing first, second, and further cycles but also in patients who received their first cycle before and after the age of 31, which represented the median age at first induction with RTX.

## Results

### Patient baseline characteristics

**Figure 1:**
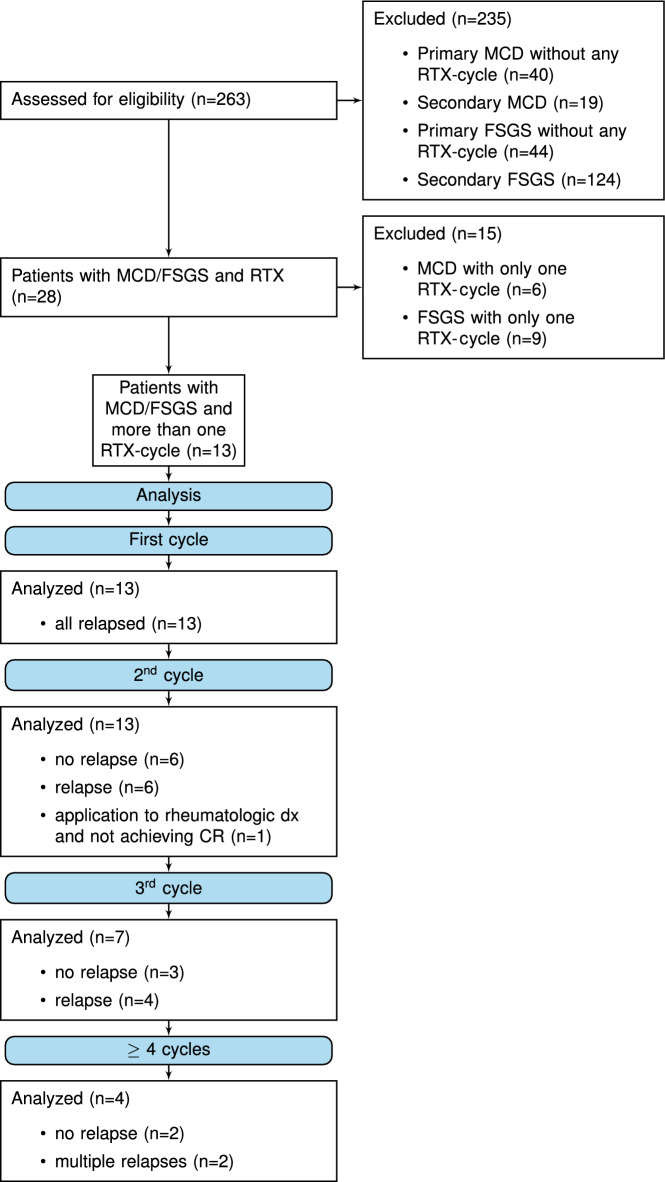
Consort flow diagram.

We identified 263 patients with MCD or FSGS. Patients with renal transplantation or secondary disease were excluded. 112 patients were treated for primary FSGS or MCD during this 10-year period. Eighty-four (75%) were treated without RTX and 28 adult patients had been treated with RTX (see Fig. 1). Of those, 15 patients had received only one cycle of RTX. Six (40%) patients showed an improved disease status, 7 (47%) remained in the same disease status and the remaining 2 (13%) patients were lost to follow-up at month 6 (*p* = 0.06).

The other 13 patients had received more than one cycle and were included in this study. Ten patients (77%) were diagnosed with MCD and three (23%) with FSGS. Analog to the pediatric classification, 15% of patients were each clinically classified as infrequently relapsing nephrotic syndrome (IRNS) or frequently relapsing nephrotic syndrome (FRNS), 46% as steroid-dependent nephrotic syndrome (SDNS), and 23% as steroid-resistant nephrotic syndrome (SRNS)^14^.

The median age at diagnosis was 11 years (IQR 7–46). The median age at the first cycle of therapy was 31 years (IQR 18–49). All patients had received at least steroids and CNI as previous therapies. Additionally, 9/13 (69%) had been treated with MMF, 3/13 (23%) with CYC, and 2/13 (15%) had been treated with various other immunosuppressants reflecting a population in which standard therapy and combinations of aforementioned drugs had failed.

Baseline creatinine at the administration of RTX was 0.98 mg/dL (IQR 0.71–1.25 mg/dL) with an eGFR of 93 mL/min/1.73$\hbox {m}^{2}$ (IQR 70–102). The eGFR was calculated with the FAS-formula to estimate GFR in both pediatric and adult patients^45^. Fifty-four percent were classified as CKD stage 1, 27.3% as stage 2 and each 9.1% as CKD stage 3 and 4. Patient specifics are detailed in Table 1.

**Table 1:** Patient characteristics.

Total number of patients	n = 13
Disease, n (%)	MCD 10/13 (77%)
	FSGS 3/13(23%)
Classification, n (%)	
IRNS	2/13 (15%)
FRNS	2/13 (15%)
SDNS	6/13 (46%)
SRNS	3/13 (23%)
Previous therapies	
Steroids	13/13 (100%)
CNI	13/13 (100%)
MMF	9/13 (69%)
Cyclophosphamide	3/13 (23%)
Other	2/13 (15%)
Gender female/male	5 (38%) / 8 (62%)
Age at diagnosis in years, median (IQR)	11 (IQR 7–46)
Age at first induction with RTX in years, median (IQR)	31 (IQR 18–49)
Baseline creatinine before RTX in mg/dL median (IQR)	0.98 (IQR 0.71–1.25)
eGFR FAS before RTX mL/min, median (IQR)	93 (IQR 70–102)
CKD stage (FAS)	
Stage 1	6/13 (46%)
Stage 2	3/13 (23%)
Stage 3	1/13 (8%)
Stage 4	1/13 (8%)
Initial stage not available	2/13 (15%)

In 7/13 patients a whole exome sequencing was performed. In these 7 patients no pathogenic mutation was detected. Six (46%) of the patients had received only 2 cycles of RTX, 3 (23%) received 3 cycles, and 2 (15%) received 4 cycles. The remaining 15% were two patients who had received 6 and 8 cycles, respectively (see Fig. 1).

A total of 43 cycles of RTX were administered. Of these, the first and second cycle were mandatory for inclusion and accounted for 26 cycles. Seven of 43 cycles were attributed to a third readministration, 4 for a fourth readministration, 2 each for fifth and sixth and 1 each for seventh and eighth readministration (see Table 2). The median dose per cycle was 2000 mg RTX [IQR 1000–2000 mg]. This did not differ between the number of cycles. Most inductions were indicated to treat a relapse with full manifestation of nephrotic syndrome. Six administrations occurred in the setting of increasing proteinuria in the subnephrotic range but did not meet the criteria for relapse. One initial administration of RTX was in a CNI-dependent frequently relapsing patient who had developed CNI-toxicity and required alternative immunosuppression. The patient relapsed later and received a reinduction with RTX.

**Table 2:** Treatment and relapse rates.

Administered RTX cycles	
Total	43
1st cycle	13/43
2nd cycle	13/43
3rd cycle	7/43
Four or more cycles	10/43
Follow up duration in months, median (IQR)	110 (IQR 81–135)
Number of induction therapies per patient, median (IQR)	3 (IQR 2–4)
Relapse rate after cycle, n (%):	
Relapse after 1st cycle	12/13 (92%)
Relapse after 2nd cycle	7/13 (54%)
Relapse after 3rd cycle	4/7 (57%)
Relapse after 4th cycle	2/4 (50%)
Relapse after 5th to 8th cycle	5/6 (83%)
Median relapse-free survival in months after RTX cycle	
First	17
Second	51
Subsequent	21

### Overall efficacy

Considering all 43 cycles, 62% of all administrations led to a complete remission and 33% to a partial remission after 3 months. Five percent of induction therapies (n = 2) did not lead to a measurable response after 3 months. After 6 months, 72% of RTX cycles led to a complete remission, 26% to a partial remission and 2% (n = 1) to no change in disease status. Disease status after 3 and 6 months was significantly different from the baseline before RTX therapy ($p<$0.001 for both time points) while there was no statistically significant difference between months 3 and 6 (*p* = 0.55).

To compare the efficacy of repeated RTX cycles, we analyzed each cycle number separately (e.g. first cycle, second cycle, third cycle). The effect of improvement of disease status could be observed at three months for the first, second ($p<$0.001), third ($p<$0.004), and following cycles ($p<$0.001). Six months after the cycle, the disease status significantly improved in the first, second, third, and following cycles ($p<$0.001 for each timepoint). Conversely, comparing the responses at comparable timepoints of all cycles (e.g. month 3 for cycle 1,2,3, and following) did not show a significant difference in the quality of response to treatment between cycles.

A robust reduction of proteinuria before RTX therapy from initially 5073 mg (IQR 3508–7786 mg) to a median of 270 mg (IQR 45–860 mg) after 3 months after the RTX administration ($p<$0.001) and a median of 94 mg (IQR 30–360 mg) after 6 months ($p<$0.001) was observed for all cycles (see Supplemental Fig. S1).

Median follow-up duration after the initial administration of RTX was 110 months (IQR 81–135) with a minimum of 5.5 months after the last administration of RTX.

**Figure 2:**
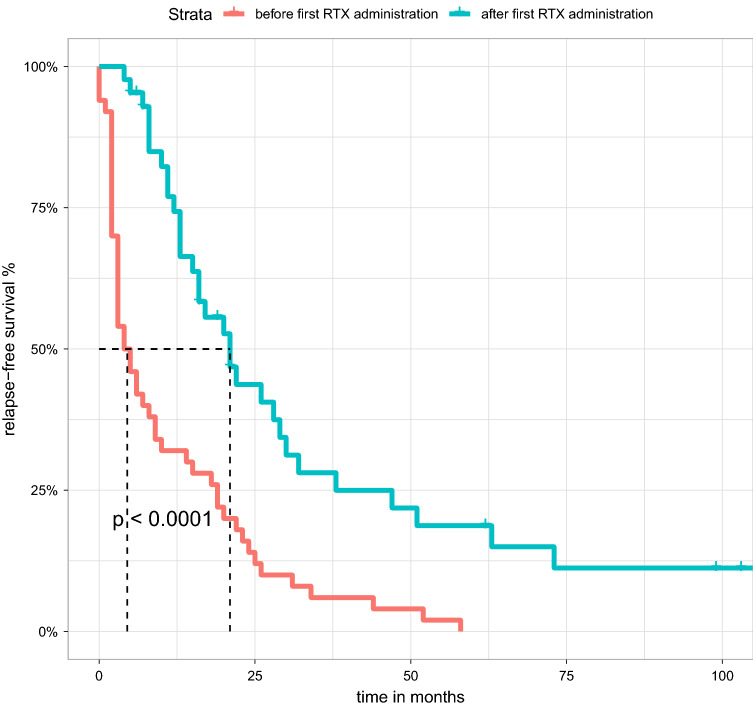
Overall median relapse-free survival improved significantly after the initiation of RTX compared to previous immunosuppression regimens. Patients who were steroid-dependent or steroid-resistant and thus directly relapsed were marked as censored at month 0.

We observed a prolongation of relapse-free survival from 4.5 months (CI 3–10 months) to 21 months (CI 16–32 months) compared to the previous immunosuppression ($p<$0.001) (see Fig. 2). Using the Kaplan-Meier estimators and Cox proportional hazard, we examined the remission duration in patients who received multiple administrations of RTX.

**Figure 3:**
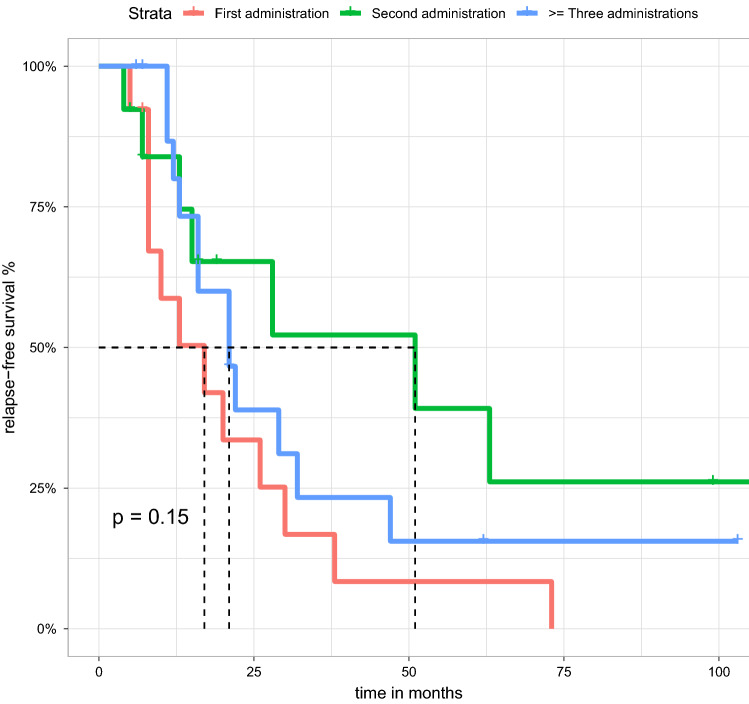
Kaplan Meier estimate showing the relapse-free survival of MCD/FSGS patients treated with RTX. There was no difference in relapse-free survival between first, second or third/subsequent courses (*p* = 0.15). Median relapse-free survival is 17 months for the first application, 51 for the second course and 21 months for subsequent courses.

We calculated the one-year relapse-free survival rate, which was 58.7% (CI 36.6–94.3%) with the first administration, 83.9% (CI 65.7–100%) with the second administration and 80% (CI 62.1–100%) with any subsequent administrations. No differences in relapse-free survival were observed with respect to the number of cycles (*p* = 0.15) (see Fig. 3).

After 6 months, 9 of 13 patients showed a similar disease status in each reinduction cycle. One patient suffered from an early relapse in the third cycle, one patient did not achieve previous disease status and remained in PR instead of previous CR. One patient with initial steroid-resistant MCD received one cycle of RTX and achieved PR. After one further cycle due to an independent rheumatoid arthritis and persistent proteinuria in the subnephrotic range, this patient improved from PR to CR and remained in remission. Individual clinical responses are shown in Supplemental Table S1, a summary is provided in Table 3.

**Table 3:** Disease status and proteinuria after RTX cycle.

Disease Status/Cycle	Overall	Second	>= Three
Before induction			
NR	35 (81.4%)	9 (69.2%)	17 (100%)
PR	7 (16.3%)	4 (30.8%)	0 (0%)
CR	1 (2.3%)	0 (0)	0 (0%)
After 3 months			
NR	2 (4.8%)	1 (7.7%)	1 (6.2%)
PR	14 (33.3%)	5 (38.5%)	3 (18.8%)
CR	26 (61.9%)	7 (53.8%)	12 (75.0%)
After 6 months			
NR	1 (2.3%)	1 (7.7%)	0 (0%)
PR	11 (25.6%)	4 (30.8%)	3 (17.6%)
CR	31 (72.1%)	8 (61.5%)	14 (82.4%)
Proteinuria in mg/d or mg/g creatinine (median/IQR)			
At induction	5073 (3508–77859)	4680 (1570–6684)	5862 (4483–11618)
After 3 months	270 (45–860)	212 (54–601)	200 (34–1654)
After 6 months	94 (31–360)	94 (83–270)	101 (32–323)

*CR* complete remission, *NR* nephrotic-range, *PR* partial remission.

Three patients maintained their previous immunosuppression, but showed a reduced frequency of relapses. In all other cases (10/13), a reduction of steroids or steroid-sparing immunosuppression was possible. The immunosuppresive regimen (removal of steroid-sparing agents or steroids) could be reduced in 5 out of 13 patients. They remained on one further steroid-sparing agent that had not maintained a longer remission before the first administration of RTX. Five out of 13 patients were free of other immunosuppressive agents.

We investigated whether renal function remained stable throughout the cycles by analyzing creatinine levels and eGFR. For both eGFR (*p* = 0.73) and creatinine (*p* = 0.53), no differences were shown between the cycles indicating no decline in renal function.

**Figure 4:**
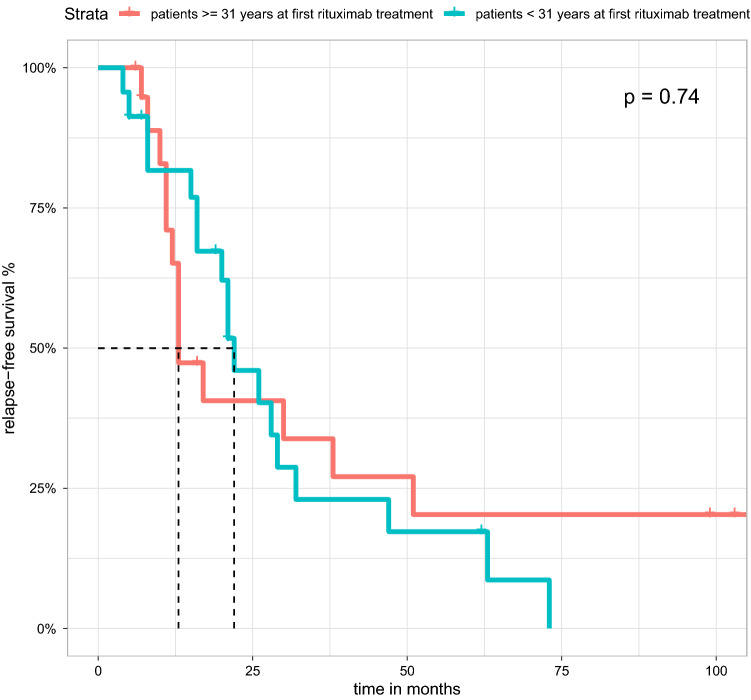
Kaplan-Meier estimate showing the relapse-free survival of MCD/FSGS patients treated with RTX stratified by age at first induction with RTX. Patients with first induction at the age $\ge$ 31 years did not show any significant difference from patients who were diagnosed before the age of 31.

In a direct comparison of patients who were first treated with RTX before the populations median age of 31 years (< 31 (n = 7) and after $\ge$ 31 years (n = 6)), no differences could be shown (*p* = 0.74) (see Fig. 4). Furthermore, we could not observe a difference in relapse-free survival comparing patients who received MMF (*p* = 0.08) or CNI (*p* = 0.47) as maintenance therapy (see Supplemental Figs. S3 and S4) or comparing patients with FSGS and MCD (*p* = 0.15, (see Supplemental Fig. S2).

### Reported side effects

In general, there were few side effects of RTX therapy. One patient developed shingles. One patient showed an infusion-associated allergic reaction that was treated with antihistamines. One patient developed mesenteric ischemia most likely attributed to hypercoagulable state in the initial phase of the nephrotic syndrome.

## Discussion

Treating frequent relapses and resistant courses of MCD and FSGS remains clinically challenging. Different therapeutic approaches have been reported. Despite the paucity of randomized clinical trials, an increasing body of literature supports the use of RTX either to achieve continuous B-cell-depletion or as intermittent reinduction therapy after relapse or in case of increasing proteinuria^29–32,35,35–37,46^.

Here, we summarize 13 patients with biopsy-proven MCD or FSGS who were treated mostly at the University Hospital of Cologne between 2010 and 2021. In our retrospective cohort, most patients had received various immunosuppressants such as steroids, CNI, MMF, and CYC mostly due to complicated disease courses before receiving RTX and thus belonged to a high-risk (and mostly multidrug-dependent to multidrug-resistant) population in MCD and FSGS with the need for alternative and long-term treatment options. Steroids, CNI, and CYC can cause long-term side effects in patients such as iatrogenic Cushing’s syndrome, diabetes mellitus, malignancy, and nephrotoxicity^47–50^. Therefore, especially in young adult patients, strategies are needed to reduce long-term sequelae and provide alternative treatment options in patients at risk of relapse without immunosuppression. RTX is generally expected to achieve at least 4 months of B-cell depletion. Thus a single administration may not be sufficient for long-term treatment^51^.

In this cohort, administration of RTX led to a significantly improved disease status with a complete remission rates of 72% and 26% for partial remission after 6 months of therapy. These remission rates are consistent with previous findings^15,21^. The median time to relapse increased from 4.5 months to 21 months after the initiation of RTX ($p<$0.001) and was comparable to pediatric sequential long-term treatment^21^.

In contrast to regimens with continuous B-cell depletion in which RTX is applied every 6 months, RTX was given only if a disease relapse occurred. The median relapse-free survival with this approach was 21 months supporting a watch-and-wait approach rather than a continuous B-cell depletion, resulting in a relapse-free survival comparable to uncomplicated MCD/FSGS courses ^52,53^. Simulating a fixed RTX administration every 6 months in our cohort resulted in 219 RTX cycles compared to 43 observed. We conclude that an individualized approach can significantly reduce RTX exposure ($p<$0.001).

With respect to the treatment costs (2x 1000mg extracted from Mabthera^©^ 1400mg Lsg 2735.44€) this approach reduced the cost by 962,874.88€ in this cohort of thirteen patients. Furthermore with respect to the COVID-19 pandemic, this strategy did not impose an unnecessary risk of immunosuppression on patients who might have suffered from a fatal course of COVID-19^54^.

Our data confirm that patients initially treated with RTX can be successfully re-treated repeatedly without diminishing efficacy. Repeated administration of RTX achieved comparable results to the first cycle after relapse. Thus, repeated cycles of RTX appear to be a viable therapeutic option for patients who have not responded well to other classes of immunosuppressants or who experience drug-related toxicities. We found no evidence of RTX resistance or differential response patterns with increasing disease duration in terms of remission duration, eGRF, and disease status after reinduction. Therefore, this strategy can be used to reduce costs and the exposure to RTX significantly without a loss of efficacy.

While in the pediatric cohort with sequential RTX treatment, adverse events occurred at a rate of 0.2 per person-years, we observed an adverse event retrospectively over a period of cumulative 110 patient-years in only 3/13 patients^21^. This was probably influenced by recall bias.

B-cell-guided treatment may be an alternative approach to reduce RTX exposure. However, stable disease can also be achieved after B-cell reconstitution^55^. RTX has demonstrated efficacy even in patients without measurable B-cells^56^. Therefore B-cell-guided therapy could lead to an too early treatment on one hand and a delayed treatment on the other hand. With the discovery of anti-nephrin autoantibodies in MCD there may be a potential biomarker for guiding therapy in anti-nephrin-antibody-positive patients^6^. Alternatively, in patients without a biomarker, weekly dipstick urine analysis can be used to self-monitor disease status. A re-treatment could be scheduled without delay reducing the risk of complications from nephrotic syndrome.

The small number of patients is an important limitation of this study. We did not find differences in relapse-free survival between FSGS and MCD or maintenance therapy with MMF or CNI in contrast to observations in the pediatric population. With respect to different age groups, comparing the older half of patients at the first administration of RTX compared to the younger half, we did not observe any age-related differences in the response to long-term treatment. Therefore, the use of RTX also seems to show a similar, non-age-dependent effect in the adult cohort.

Further limitations of our study are the retrospective nature of our study with outcome parameters reported at irregular intervals. In addition, most patients had good kidney function at the time of the first administration of RTX. A subgroup analysis of patients with marked renal impairment was not possible due to the small number of cases. Compared to prospective studies, our adverse event reports were most likely influenced by recall bias.

We cannot safely conclude that an individualized approach may not lead to an increase in adverse outcomes due to relapses of nephrotic syndrome (e.g. increase in thromboembolic events). On the other hand, maintenance therapy puts the patient at risk for infectious and malignant complications that need to be balanced with the risk of relapse of nephrotic syndrome. A refinement in the weekly monitoring of dipstick urine might be an approach to detect recurrence early. Further insights into the pathomechanism of FSGS and MCD, especially with respect to potential biomarkers such as anti-nephrin antibodies and suPAR may lead to a more individualized treatment approach and help identify patients with no or little benefit from therapy^11^. With respect to primary RTX resistance, advanced anti-CD20 antibodies have anecdotally been used in MCD and FSGS, but their role remains ill-defined in relation to RTX^57,58^. Due to the low incidence of MCD and FSGS, particularly of complicated courses and the wide variability in clinical courses, a meaningful prospective randomized trial seems hard to envision in the adult population, placing particular emphasis on the impact of real-world data and clinical registries with biosampling^38^.

## Conclusion

In conclusion, in adult patients with MCD and FSGS who require long-term treatment options with RTX, repetitive administrations of RTX were not associated with a habituation effect and led to high percentage of complete or partial remission comparable to larger pediatric studies. The renal function remained stable over the treatment peroid. This approach led to a prolongation of relapse-free survival compared to previous treatment regimens over a median follow-up duration of 110 months.

An individualized approach significantly reduced the costs and the cumulative exposure to RTX compared to continuous B-cell depletion.

The data presented here make an argument for an individualized approach to RTX maintenance therapy in complicated courses of MCD and FSGS. Due to the paucity of randomized clinical trials in the adult population, clinical registries provide an important source of information for research on rare diseases.

## Supplementary Information

Supplementary Information.

## Data Availability

The datasets used and/or analysed during the current study available from the corresponding author on reasonable request.
