# Supplementary material for: Repetitive administration of rituximab can achieve and maintain clinical remission in patients with MCD or FSGS
Source: Sci Rep. 2023 Apr 28;13:6980. doi: 10.1038/s41598-023-32576-7 (PMC10141841; doi:10.1038/s41598-023-32576-7)

Supplemental table 1

Supplemental Table S1. Supplemental Table 1 showing individual responses (CNI=calcineurin-inhibitors, CR=complete remission, CYC=cyclophosphamide, L=Levamisole, MMF=mycophenolate-mofetil, NR=nephrotic-range, PR=partial remission, ST=steroids)

Patient	Sex	Age at diagnosis	Disease	Classification	Rituximab Cycle No.	Classfication before Rituximab	Classfication 3 Months after Rituximab	Classfication 6 Months after Rituximab	Duration of Remission (months)	Serum-Creatinine	eGFR FAS (ml/min)	Previous therapies
A	m	7	MCD	SDNS	1	CR	CR	CR	26	1.16	72	ST,CNI
A					2	PR	CR	CR	19	0.82	115	MMF
B	f	10	MCD	SDNS	1	PR	CR	CR	38	0.81	93	ST, CNI,
B					2	PR	PR	CR	89	0.69	109	MMF, CYC
C	f	58	FSGS	SRNS	1	NR	PR	PR	7	3.08	19	ST, CNI,
C					2	PR	CR	CR	103	0.65	91	TNF-Blockers
D	m	35	MCD	SDNS	1	NR	PR	PR	13	0.98	99	ST, CNI
D					2	NR	PR	PR	7	0.87	111	MMF
E	m	8	MCD	IRNS	1	NR	CR	CR	73	0.95	102	ST, CNI,
E					2	PR	CR	CR	5,5	0.66	146	CYC
F	f	60	FSGS	SRNS	1	NR	PR	CR	10	1.62	35	ST, CNI, MMF
F					2	NR	CR	CR	16	1.43	40	
G	f	4	MCD	SRNS	1	NR	PR	PR	8	1.33	69	ST,CNI,
G					2	NR	PR	PR	63	0.97	94	MMF, CYC, L
G					3	NR	PR	PR	62	1.25	61	
H	m	46	FSGS	IRNS	1	NR	CR	CR	30			ST, CNI,
H					2	NR	PR	PR	7			MMF
H					3	NR	PR	PR	103			
I	m	31	MCD	FRNS	1	NR	PR	PR	17			ST, CNI
I					2	NR	PR	PR	51	1.20	70	MMF
I					3	NR	PR	PR	6			
J	f	2	MCD	SDNS	1	PR	CR	CR	20	0.61	150	ST, CNI,
J					2	NR	CR	CR	28	0.67	112	MMF
J					3	NR	CR	CR	21	0.63	119	
J					4	NR	CR	CR	29	0.76	99	
K	m	7	MCD	SDNS	1	PR	CR	CR	8	0.60	102	ST,CNI
K					2	NR	NR	NR	4	0.56	113	MMF
K					3	NR	NR	CR	16	0.53	119	
K					4	NR	CR	CR	21	0.99	98	
L	m	59	MCD	SDNS	1	NR	CR	CR	8			ST, CNI
L					2	NR	CR	CR	13	1.49	42	
L					3	NR	CR	CR	12			
L					4	NR	CR	CR	11	1.70	41	
L					5	NR	NaN	CR	11	1.58	44	
L					6	NR	CR	CR	13	2.74	25	
M	m	11	MCD	FRNS	1	NR	PR	CR	5	0.59	107	ST, CNI,
M					2	NR	CR	CR	15	0.5	131	MMF
M					3	NR	CR	CR	47	0.57	136	
M					4	NR	CR	CR	22	0.75	126	
M					5	NR	CR	CR	21	0.85	114	
M					6	NR	CR	CR	32	0.92	105	
M					7	NR	CR	CR	16	0.91	106	
M					8	NR	CR	CR	7	1.04	96	

Supplemental figure 1

Supplemental Figure S1. Level of proteinuria overall and for individual cycle

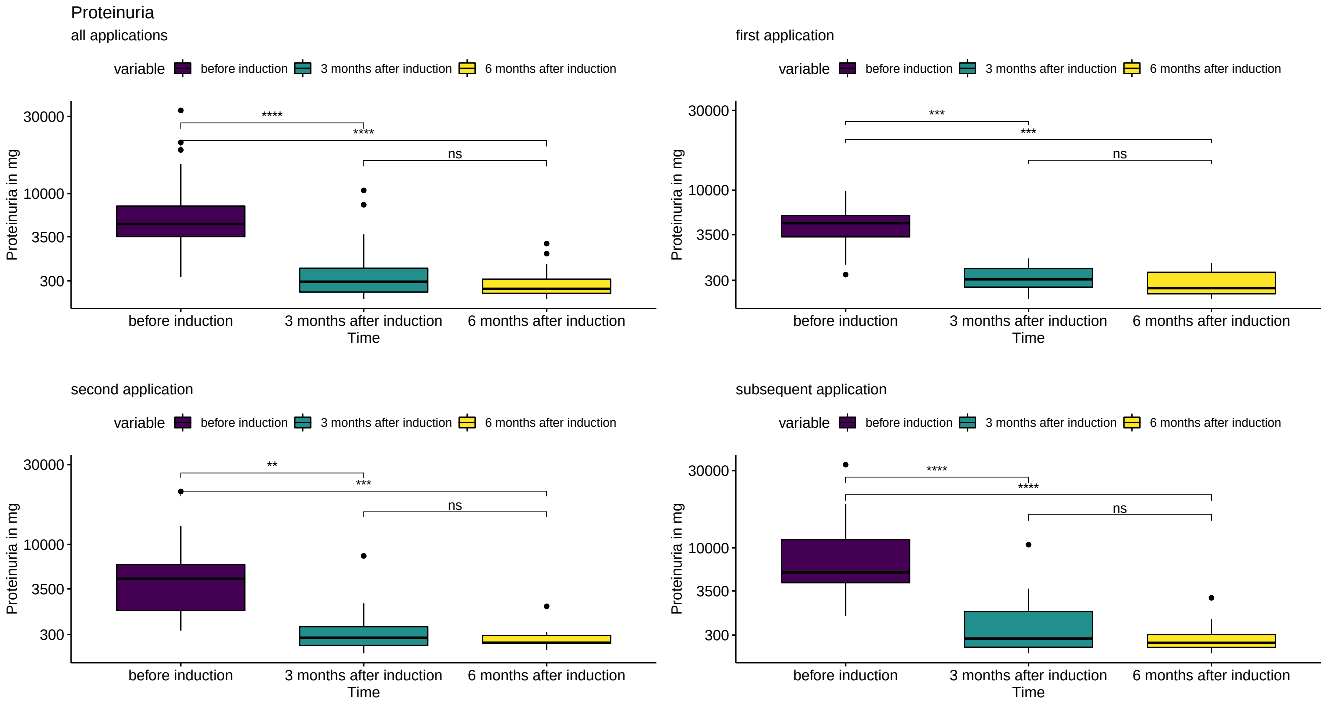

Supplemental figure 2

Supplemental Figure S2. Relapse-free survival by histology

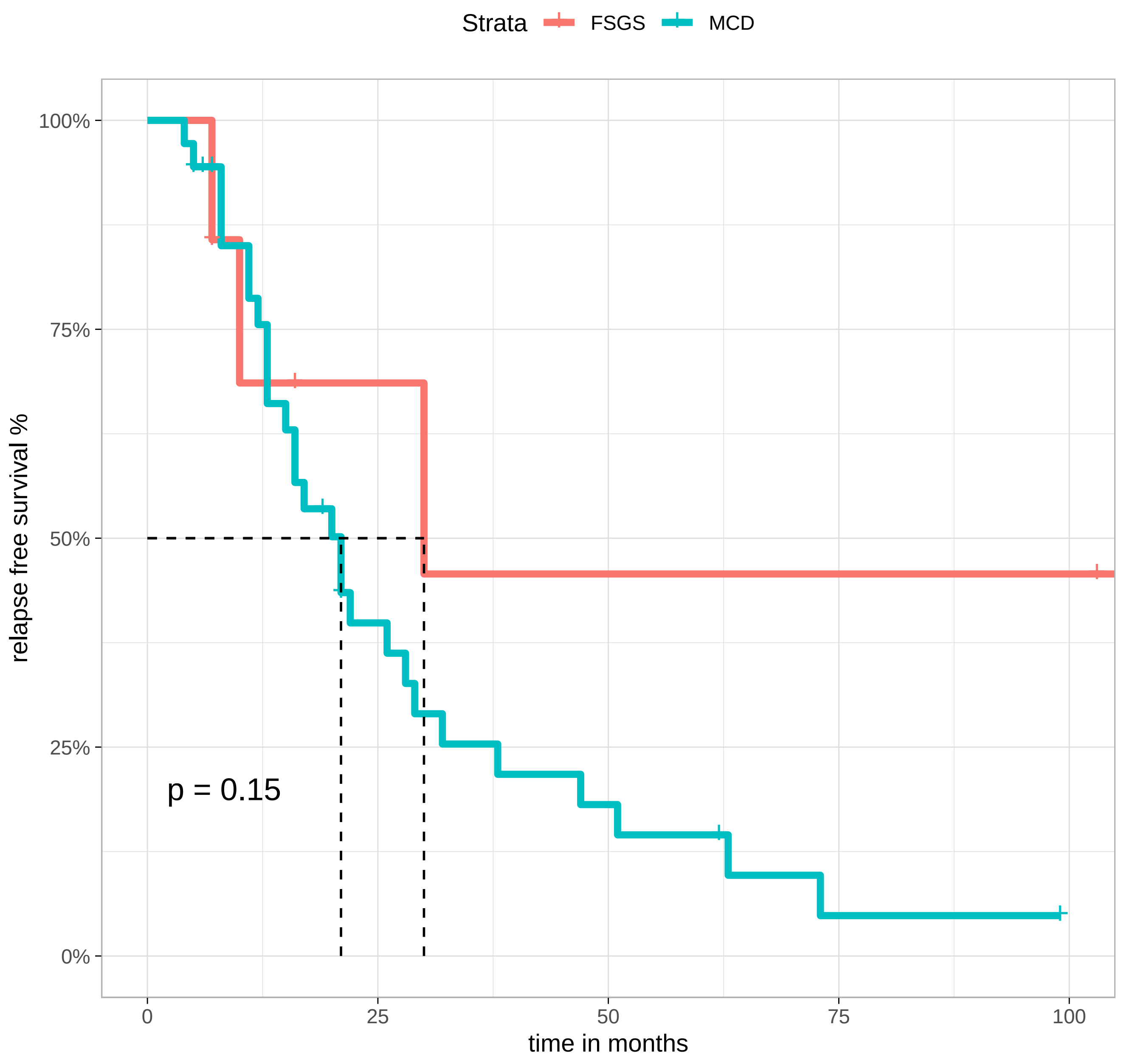

Supplemental figure 3

Supplemental Figure S3. Relapse-free survival after rituximab based on MMF-maintenance therapy

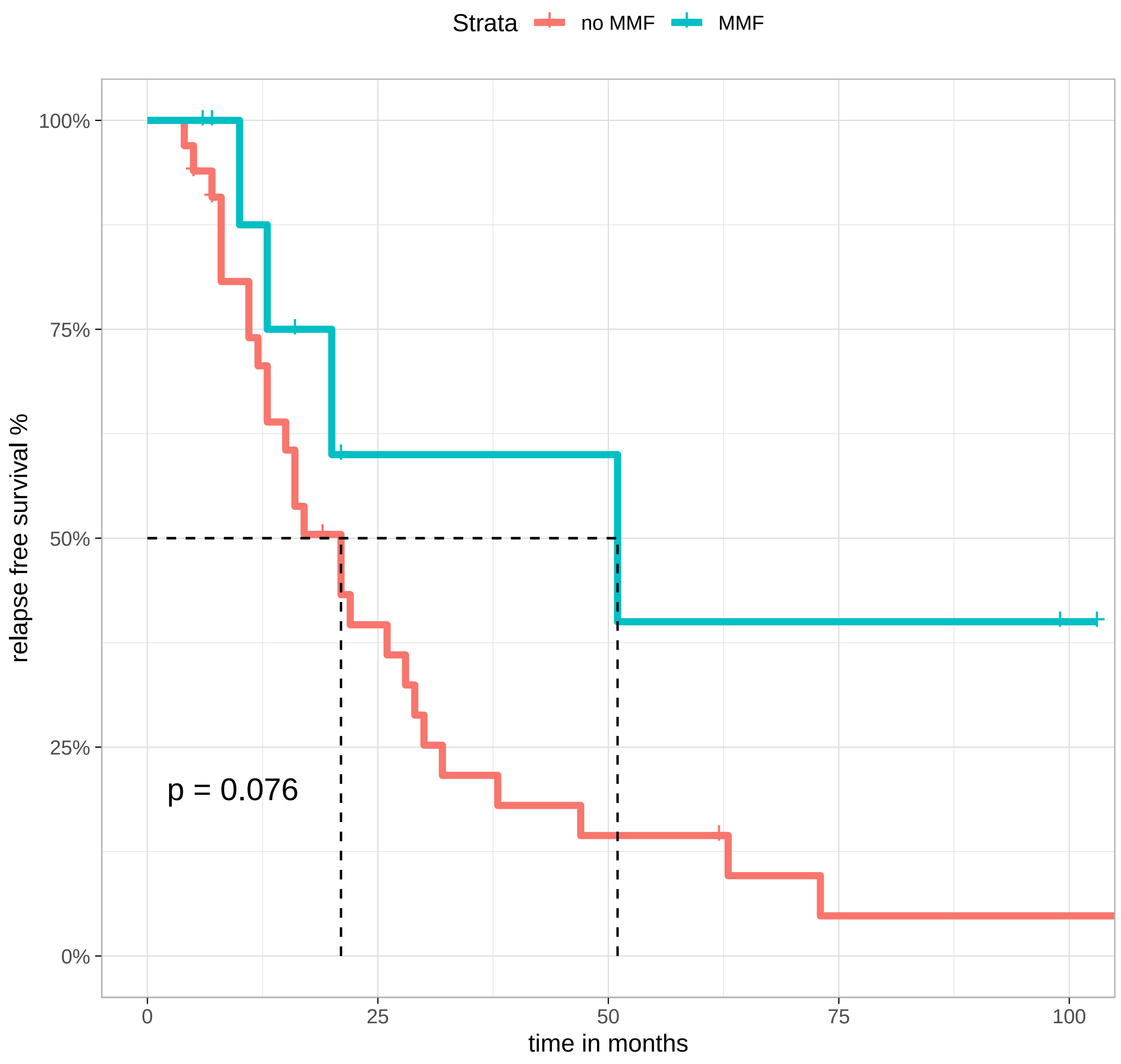

Supplemental figure 4

Supplemental Figure S4. Relapse-free survival after rituximab based on CNI-maintenance therapy

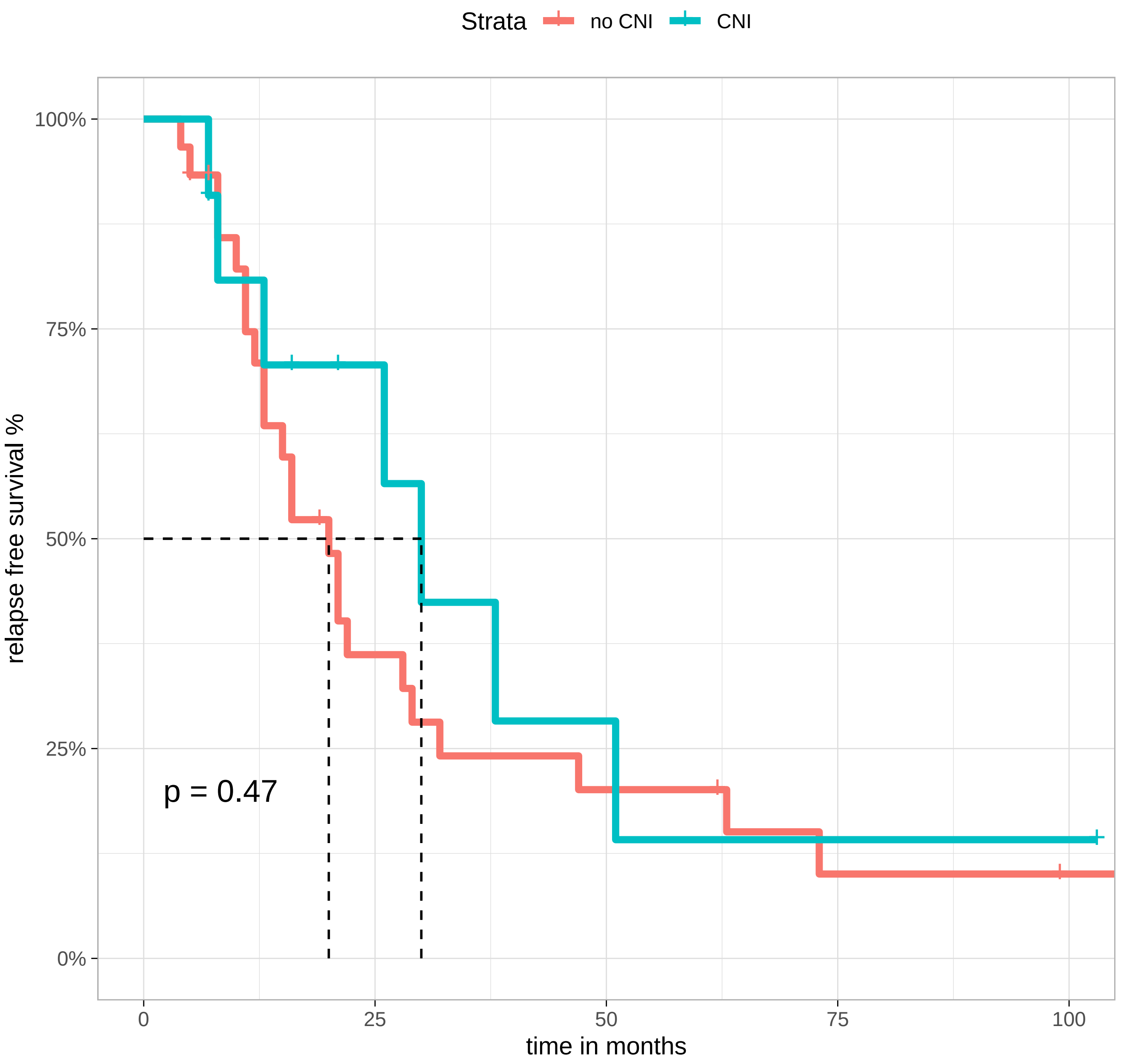

Supplement: Supplementary file 1 — Supplementary Information. [file 41598_2023_32576_MOESM1_ESM.pdf]
